# Comparative Study of Hemodynamics Electrolyte and Metabolic Changes During Prone and Complete Supine Percutaneous Nephrolithotomy

**DOI:** 10.5812/numonthly.4099

**Published:** 2012-09-24

**Authors:** Hosein Khoshrang, Siavash Falahatkar, Sara Ilat, Manzar Hossein Akbar, Maryam Shakiba, Alireza Farzan, Nadia Rastjou Herfeh, Aliakbar Allahkhah

**Affiliations:** 1 Urology Research Center, Razi Hospital, Guilan University of Medical Sciences, Rasht, IR Iran

**Keywords:** Electrolyte, Prone Position, Supine Posion, Percutaneous Nephrolithotomy

## Abstract

**Background:**

Nowadays Percutaneous Nephrolithotomy (PCNL) is performed in prone and supine positions. Physiologic solutions should be used to irrigate during PCNL. Irrigation can cause hemodynamic, electrolyte and acid-base changes during PCNL.

**Objectives:**

The current study aimed to compare the electrolyte, hemodynamic and metabolic changes of prone and complete supine PCNL.

**Patients and Methods:**

It was a randomized clinical trial study on 40 ASA class I and II patients. Twenty of patients underwent prone PCNL (Group A) and the other twenty underwent complete supine PCNL (Group B). The two groups received the same premedication and induction of anesthesia. Blood pressure (systolic, diastolic and mean) and pulse rate were recorded before, during and after anesthesia and Hb, Hct, BUN, Cr, Na, and K were also measured before and after operation in the two groups. The volume of irrigation fluid, total effluent fluid (the fluid in the bucket and the gazes) and volume of absorbed fluid were measured.

**Results:**

There were no significant differences in Na, K, BUN, Cr, Hb and Hct between the two groups. Absorption volume was significantly different between the two groups (335 ± 121.28 mL in group A and 159.45 ± 73.81 mL in group B, respectively) (P = 0.0001). The mean anesthesia time was significantly different between the two groups (P = 0.012). There was a significant difference in bleeding volume between supine and prone PCNL (270.4 ± 229.14 in group A and 594.2 ± 290 in group B, respectively) (P = 0.0001). Mean systolic blood pressure during operation and recovery was 120.2 ± 10.9 and 140.7 ± 25.1 in group B, and 113.4 ± 6.4 and 126.2 ± 12.7 in group A, respectively. Systolic blood pressure between the two groups during operation and recovery was significantly different (P = 0.027 and P = 0.022, respectively). Mean diastolic blood pressure in supine group during operation and recovery was 80.53 ± 7.57 and 95.75 ± 17.48, and 73.95 ± 3.94 and 83.4 ± 12.54 in prone group, respectively. Diastolic blood pressure was significantly different between the two groups. It was 80.55 ± 7.57 and 95.75 ± 17.48, respectively during operation and recoveryin the supine group and 73.95 ± 3.94 and 83.4 ± 12.54 in the prone group, respectively (P = 0.001 and P = 0.014, respectively), but there was no significant difference between the pulse rate mean value of the two groups.

**Conclusions:**

The electrolyte and metabolic changes were not significantly different between the two groups, and although fluid absorption in prone group was more than that of the complete supine group, there was no significant difference between the two groups. Considering advantages of complete supine PCNL such as less hemodynamic changes (less hypotension, less fluid absorption and less duration of operation) this kind of PCNL was recommended.

## 1. Background

Percutaneous nephrolithotripsy (PCNL) is a common technique to treat kidney stones and is also used to fragment and remove the calyx and pelvic stones (1-5). Nowadays to manage large stones, stones resistant to fragmentation, or stones with an abnormal anatomy in kidney, PCNL is preferred (6-8).

Generally, the advantages of PCNL are less mortality rate, less pain after operation, quick improvement after operation and less scar formation. PCNL is usually performed in the prone position but the complete supine position (csPCNL) has potential advantages compared with the prone position. The lateral and some modified supine positions were reported safe in high-risk patients and also all the other cases. The patients in csPCNLwere placed at the bed edge. There was no rolled tower on the flank and no change in leg position in csPCNL. This endoscopic technique (csPCNL)which needs continuous irrigation can result in serious complications (5). One of the most important complications is extravasationof large amount of irrigation fluid to retroperitonealspace that increases the likelihood of septic complications (9-12).

There were few surveys on hemodynamic, electrolyticand acid-base changes due to PCNL, which suggested different ideas (8). In some studies electrolytic changes due to PCNL, showed hyponatremia and metabolic acidosis other than hypertension (3, 13). To avoid complications due to absorption of fluid without electrolytes, normal saline is the fluid which is commonly used for irrigation (3, 5). Manipulation under X-Ray or endoscopy, by continuous open flowing systemcan also be usedto prevent electrolytic imbalance. If the difference between inflow and outflow fluid is more than 500 mL, operation should be stopped and a nephrostomy tube must be applied, and electrolytes measurement is also necessary. Ethanol monitoring can also help to evaluate absorption volume and direction detection (14).

## 2. Objectives

Considering the limited number of studies on hemodynamic, metabolic and electrolyte changes due to PCNL and lack of studies on comparison of electrolytic, hemodynamic and metabolic changes between the supine and the prone PCNL, it was decided to analyze the effects and the fluid absorption levels between the two methods of operation.

## 3. Patients and Methods

In the clinical trial done in a period of 6 months on 40 patients with ASA class I, and II, who had undergone prone or complete supine PCNL, the subjects were divided in two groups (20 patients in each groups) by blocked randomization method. Patients with hypertension, heart failure, renal failure and those who had undergone any kind of medical therapy which could affect hemodynamic and electrolyte status, were not included in the study. Inclusion criteria were having one or more stones > 2 cm which could be removed by a percutaneous surgery and no contraindications for the prone position. Exclusion criteria were kidney anomalies, uncontrolled coagulopathies, pregnancy, immunodeficiency, ASA class III and IV and age < 10 year old.

Before the surgery, systolic, diastolic and mean blood pressure and pulse rate, Hb, HCT, BUN, Cr, Na and K were assessed and measured in a blood sample. Anesthesia was induced by Sodium Thiopental (5 mg/kg), Atracorium (0.6 mg/kg) and Fantanyl (2 µg/kg), and maintained with halothane 0.5%, N2O + O2 (50:50) and Atracorium (0.2 mg/kg) every 30 minutes. At the end of the procedure, neuromuscular blockage was reversed by Neostigmin 0.04 mg/kg and Atropine 0.02 mg/kg.

Ringer was used as an intravenous fluid in all patients. All of the patients got dextrose saline as maintenance fluid therapy after the operation. If there was more than 20% hypotension from the baseline, Normal saline or Ringer fluid were replaced. Irrigation fluid was Glycine. Total volume of irrigation fluid which was used and total effluent fluid (the fluid in the grading bucket and the number of drench gazes) were measured and the difference between them was taken as the absorbed fluid volume.

The second blood sample was taken 6 hours after the operation to measure blood hemoglobin (Hb), hematocrite (HCT), blood urea nitrogen (BUN), creatinine (Cr), Na and K. Blood pressure and pulse rate were measured before anesthesia, during induction and intubation period, every 5 minutes during maintenance of anesthesia and after anesthesia with ECG non invasiveblood pressure (NIBP) and by the use of a pulse oxymeter (model: B5 – SNTI/E_2_/M/C manufactured by Pooyandegane Rah Saadat company) the level of saturation was monitored.

The size of stones were also evaluated by kidney ureter bladder radiography (KUB) and sonography. All data were analyzed by paired t-test, Turkey’s and independent t-test and P value < 0.05 was considered significant.

## 4. Results

From 20 patients in the prone group, 6 patients (30%) were female and 14 patients (70%) were male. In csPCNL group 12 patients (60%) were male and 8 patients (40%) were female. Mean age of patients was 46.07 ± 10.43 (range 23-70) years old. The mean anesthesia duration in the supine group was 110.5 ± 20.76 min and in the prone group it was 137.25 ± 39.31 min, and there was no significant difference between the two groups (P = 0.12). In the supine group, the mean Hb was 13.46 ± 1.65 mg/dLand the mean Hb after operation was 11.97 ± 1.61 mg/dL.The current studyindicateda significant difference in mean levelof Hb and HCT in the supine group before and after operation (P = 0.0001, P = 0.0001).

In the prone group the mean Hb before operation was 13.95 ± 1.68 mg/dL and after the operation was 11.94 ± 1.931 mg/dL, which indicated that there was significant difference between Hb and HCT levels before and after operation (P = 0.0001, P = 0.0001) but there was no significant difference in comparison of Hb and HCT before and after the operation between the two groups. Volume of bleeding during the operation in the supine group was 270.4 ± 229.14 cc and in the prone group 594.2 ± 290.74 cc. There was a significant difference between the bleeding mean volume, during the supine and the prone PCNL.

The mean volume of irrigation fluid during the operation was 196.5 ± 4.12 cc in the supine group and 197 ± 5.26 cc in the prone group. There was no significant difference between the two groups (P = 0.0753) in this regard. The volume of absorbed fluid during the operation was 159.45 ± 73.8 in the supine group and 355 ± 121.28 in the prone group. There was a significant difference between the two groups (P = 0.0001) in this regard.

Mean systolic blood pressure during the operation and the recovery was 120.2 ± 10.9 and 140.7 ± 25.1, in the supine group and 113.4 ± 6.4 and 126.2 ± 12.7, in the prone group, respectively. There was a significant difference in systolic blood pressure of the two groups during the operation and recovery (P = 0.027, P = 0.022, respectively). Mean diastolic blood pressure during operation and recovery was 80.55 ± 7.57, 95.75 ± 17.48, in the supine group, and 73.95 ± 3.94, 83.4 ± 12.54, in the prone group, respectively. There was also a significant difference in diastolic blood pressure of the two groups during the operation and recovery (P = 0.001, P = 0.014) (Table 1). Finally, mean heart rate was not significantly different between the two groups (Table 2).

**Table 1:** Comparison of Mean Systolic and Diastolic and Mean Blood Pressure in Different Time Episodes in Patient’s Prone (Group A) and Supine Position (Group B)

variable	Time Episodes	Group	Mean	SD	P value
**Systolic**					
	Pre induction				0.281
		supine	138.3	14.3	
		Prone	133.5	13	
	Induction				0.812
		supine	113.9	15.2	
		Prone	114.9	9	
	Operation				0.022
		supine	120.2.	10.9	
		Prone	113.4	6.4	
	Extubation				0.544
		supine	126.6	21.1	
		Prone	123.5	8.1	
	Recovery				0.027
		supine	140.7	25.1	
		Prone	126.2	12.7	
**Mean**					
	Pre induction				0.204
		supine	115.1	14.98	
		Prone	110	9.15	
	Induction				0.495
		supine	92.9	14.53	
		Prone	95.55	9.15	
	Operation				0.184
		supine	98.4	10.47	
		Prone	94.75	5.84	
	Extubation				0.609
		supine	102.1	18.6	
		Prone	104.45	8.13	
	Recovery				0.039
		supine	116.2	16.68	
		Prone	107	9.51	
**Diastolic**					
	Pre induction				0.261
		supine	88	8.02	
		Prone	85	8.57	
	Induction				0.345
		supine	77.35	13.52	
		Prone	74	7.91	
	Operation				0.001
		supine	80.55	7.57	
		Prone	73.95	3.94	
	Extubation				0.812
		supine	83.8	13.11	
		Prone	83	7.21	
	Recovery				0.014
		supine	95.75	17.48	
		Prone	83.4	12.54	

**Table 2:** Comparison of Different Between Lab Data Before and After Surgery in Patients With Supine (Group B) and Prone Position (Group A)

	Group	Time	Mean	SD	P value
**BUN**					
	Supine				0.009
		Before	15.9	5.23	
		After	14.2	4.94	
	Prone				0.505
		Before	14.7	4.65	
		After	14	3.69	
**Cr**					
	Supine				0.815
		Before	1.01	0.31	
		After	1	0.29	
	Prone				0.644
		Before	0.9	0.4	
		After	0.93	0.3	
**Na+**					
	Supine				0.211
		Before	138.65	3.63	
		After	139.8	4.93	
	Prone				0.201
		Before	139.1	3.16	
		After	139.95	3.42	
**K+**					
	Supine				0.918
		Before	4.37	0.38	
		After	4.36	0.25	
	Prone				0.124
		Before	4.26	0.44	
		After	4.44	0.5	

The mean level of BUN before and after the operation was 15.9 ± 5.23 and 14.2 ± 4.94 in the supine group, and 14.7 ± 4.65 and 14 ± 3.69 in the prone group, respectively. The Cr level before and after the operation was 1.01 ± 0.31 and 1 ± 0.29, in the supine group , and 0.9 ± 0.4 and 0.93 ± 0.3 in the prone group, respectively.

Mean Na level before and after the operation was 138.65 ± 3.63 and 139.8 ± 4.93 in the supine group and 139.95 ± 3.43 in the prone group, respectively. Mean K level before and after the supine PCNL was 4.37 ± 0.38 and 4.36 ± 0.25 in the supine group, and 4.26 ± 0.44 and 4.44 ± 0.5 in the prone group, respectively.

There was no significant difference in BUN, Cr, Na, K levels between the two groups (the prone and the supine) before and after the operation (Table 3). Mean stone diameter in the supine group was 26.32 ± 9.15 mm and in the prone group was 26.8 ± 5.78 mm and there was no significant difference between the two groups (P = 0.846) regarding the mean stone diameter.

**Table 3:** Comparison of Mean Heart Rate in Different Time Episodes in Patients Prone (Group A) and Supine Position (Group B)

Time Episodes	Group	Mean	SD	P value
**Pre induction**				0.654
	Supine	80.3	12.8	
	Prone	82	9.5	
**Induction**				0.589
	Supine	72.1	12.8	
	Prone	73.8	5.4	
**Operation**				0.861
	Supine	70.5	7.3	
	Prone	70.9	7	
**Extubation**				0.086
	Supine	72.6	9	
	Prone	77.3	7.8	
**Recovery**				0.799
	Supine	80.4	14.5	
	Prone	81.5	12.5	

## 5. Discussion

In the current study the electrolyte, hemodynamic and metabolic changes in the prone and complete supine PCNL were compared. In the study of Mohta et al. there was no significant change in mean heart rate and arterial blood pressure before and after irrigation (the irrigation fluid was normal saline) (3).

Also Koroglu et al., couldn’t find significant changes in blood pressure, heart rate and central venous pressure before and after irrigation (13). In the current study, systolic and diastolic blood pressure during the operation and in the recovery room and mean blood pressure in the recovery room decreased considerably in the prone group in comparison to the supine group. Considering that in prone position, pressure on abdomen can decrease venous return by compressing the abdominal veins, maybe a decrease in venous return is the reason of hypotension during the operation in the prone position. Although absorbed fluid was more in the prone group, probably it was not enough to improve hemodynamic imbalance which occurred during the operation.

In Mohta and Koroglu’s studies there was no significant change in electrolyte levels (Na and K) (3, 13). In another study ,it was found that after irrigation by distilled water there was a significant change in Na but not in K (8). In the current study, changes in Na and K levels before and six hours after the operation were not significant between the two groups and relationship between Na, K and the volume of used and absorbed fluid were not considerable.

Mohta and Koroglu found no significant difference between BUN and Cr levels before and after the operation, but in Kilic’s study, Cr level significantly increased immediately after PCNL, but on the following day of the operation it decreased in comparison to its preoperation level. Changes of BUN level were not significant (2, 12,13). In the current study, in the supine group, BUN levelchanged significantly after the operation in comparison to its level before operation, but comparing BUN and Cr levels in the two groups, no significant difference was found. It was not related to volume of used and absorbed fluid either.

In a study on 80 patients who underwent PCNL (40 patients underwent csPCNL and 40 patients underwent prone PCNL), blood transfusion was needed because of the bleeding volume, there was no significant difference between the supine and the prone groups (15). In another study, 28 patients underwent PCNL and irrigation was performed by isotonic solutions such as manitol , in which, bleeding during operation was a warning sign and was an effect of the irrigation fluid used (16).

In the current study, bleeding during the operation was significantly higher in the prone group in comparison to the supine group. In the supine group, one case (5 %) and in prone group 3 cases (15 %) needed transfusion but the difference was not significant. The bleeding was detected from surgical field, a drop of Hb and HCT pri-operative occured. Considering that the same irrigation fluid was used both groups and there was no significant relationship between the used fluid and bleeding in the two groups, may be one cause for more bleeding in the prone group was the more fluid absorption in this group (16). Intraoperative bleeding seems to be associated with intraoperative hypothermia, during the surgery and the volume of fluid intake (17).

Venous return can be impaired because of the pressure on the abdomen through the abdominal veins in the prone position (12). In another study, there was a significant relationship between the duration of the operation in the prone and the complete supine PCNL which was significantly lower in the supine position (P value < 0.0001) (15).

According to the above studies, there was a significant difference between the duration of the operation in the prone and the complete supine PCNL which was significantly lower in csPCNL (P value< 0.012). The duration of csPCNL was lower than the prone PCNL and this can be justified by the time spent to change from supine position to prone position in the prone group. The volume of absorbed fluid during operation was 159.45 ± 73.8 in supine group and 355 ± 121.28 in prone group which indicated a significant difference between the two groups (P = 0.0001). The amount of absorbed fluid depends mostly on the irrigant pressure and the length of the procedure (18, 19).

Considering the results of the current study and some other related studies, it can be concluded that the complete supine PCNL was more advantageous according to its less hemodynamic changes (less hypotension), less fluid absorption, lower duration of operation, less bleeding and need for transfusion, better access to urethra, less manipulation of the patient, better control of airways during the operation, and possibility of simultaneous PCNL and urethroscopy.
